# Enhancing Neural Efficiency in Competitive Golfers: Effects of Slow Cortical Potential Neurofeedback on Modulation of Beta Activity—An Exploratory Randomized Controlled Trial

**DOI:** 10.3390/neurosci6040104

**Published:** 2025-10-14

**Authors:** Eugenio Lizama, Luciana Lorenzon, Carolina Pereira, Miguel A. Serrano

**Affiliations:** 1 Sport Brain Center, 1000 Pembroke Rd, Hallandale Beach, FL 33009, USA; eugenio.lizama@bpgchile.cl; 2 Centro Italiano di Neurofeedback, Viale IV Novembre, 83, 31100 Treviso, Italy; info@cinb.it; 3 Centro de Entrenamiento Neuropsicológico, Avenida Italia 1883, Santiago de Chile 7770385, Chile; cpereirahinke@gmail.com; 4 Department of Psychobiology, Universitat de València, 46022 València, Spain

**Keywords:** slow cortical potentials, neurofeedback, neural efficiency, beta oscillations, golf

## Abstract

Background: Neural efficiency theory proposes that expert athletes optimize brain resource allocation and functioning. Beta band oscillations are associated with attention, motor preparation, and emotional control, reflecting adaptive patterns of reduced cortical energy expenditure (absolute power) and greater temporal precision (peak frequency). Slow cortical potential (SCP) neurofeedback has emerged as a method to train voluntary cortical regulation, yet its application in sports—particularly in precision-demanding disciplines such as golf—remains underexplored. The aim of this study was to evaluate the effects of SCP neurofeedback on beta band activity in competitive golfers. Methods: Forty-two golfers were randomly assigned to either an intervention group (*n* = 21), which completed 16 SCP neurofeedback sessions (2560 trials), or a control group (*n* = 21). SCP activity was measured during activation and deactivation trials, while EEG beta oscillations were analyzed in terms of peak frequency and absolute power at C3, O2, F8, and T5. These sites were chosen for their relevance to golf: C3 (motor execution), O2 (visual processing), F8 (inhibitory and emotional control), and T5 (visuospatial integration). Results: The intervention group showed significant increases in positive SCP trials, reflecting improved voluntary cortical inhibition. Peak frequency increased in Beta 1 (C3) and Beta 2 (O2), while absolute power decreased at F8 and T5, which seems to indicate a reduced cortical overload and enhanced visuospatial integration. Conclusions: SCP neurofeedback modulated beta activity in golfers, enhancing neural efficiency and supporting its potential as an innovative tool to optimize performance in precision sports.

## 1. Introduction

Optimal performance is defined as the ability of an individual to execute an athletic activity with extraordinary outcomes under specific conditions that challenge their mental capacities [1], technical training level, and physical conditions [2]. The complex relationship between the nervous system and sports performance has been a focal point in applied psychology and neuroscience, leading to innovative approaches aimed at enhancing skills and performance. Several authors have highlighted that attentional control of relevant stimuli and motor execution [3], decision-making [4], and, finally, proper affective regulation [5] are essential for achieving successful performance.

In golf, a sport that requires not only physical skill but also a high degree of cognitive processing, strategic planning, sensorimotor control, and emotional regulation, which are critical for maintaining concentration and precision while managing stress in competitive scenarios [6]. The ability to perform tasks with optimal physical energy and the minimal necessary brain activity, known as neural efficiency [7], has been directly linked to superior performance, since athletes with higher neural efficiency display better focus, faster decision-making, and more effective motor control [8].

The concept of neural efficiency and its impact on sports performance is not new [7], as it is well established that it is intrinsically associated with the minimal consumption of energy in perceptual–cognitive–motor processes, as well as the suppression of irrelevant preparatory motor and cognitive processes (i.e., neuromotor noise). This is known as the Neural Efficiency Hypothesis, characterized by focusing on the central elements of the task while inhibiting irrelevant processes [9]. This occurs thanks to a well-developed internal model and memory representation that is permanently contrasted with the task, together with the specific and functional coordination of movement programming processes during motor preparation [10,11,12]. However, its specific application and measurement in golf—a sport that uniquely combines physical precision and mental sharpness—is less understood, and the integration of neurofeedback training as a method to enhance neural efficiency in golfers represents a novel area of exploration [13].

Neurofeedback allows real-time visualization of brain activity and the training of self-regulation of brain functions to improve sports performance [14]. In golf, neurofeedback protocols such as SMR (12–15 Hz, C3/Cz/C4) have been successfully used to improve motor response precision [15,16]. Likewise, the theta/beta protocol (↓4–7 Hz; ↑15–20 Hz) is used to optimize sustained attention and inhibitory control [15,16]. Overall, systematic reviews and meta-analyses report improvements in reaction time, precision skills, and motor performance in athletes and healthy adults [16,17], yet heterogeneity persists across studies; therefore, standardized protocols/parameters and longitudinal follow-ups are recommended, in line with the CRED-nf checklist [18].

Research in this field has demonstrated that slow cortical potential (SCP) training via neurofeedback can lead to significant improvements in concentration and stress reduction, ultimately promoting optimal performance [19,20].

SCP is a neurophysiological phenomenon that reflects gradual electrical shifts (negative or positive) in neuronal membrane polarization, which can occur approximately 500 ms after stimulus presentation and last from several hundred milliseconds up to several seconds [21,22]. SCPs provide a unique perspective on cortical excitability and inhibition dynamics during the preparation and execution of cognitive and motor tasks [23]. These potentials differ from oscillatory activity (e.g., delta, theta, alpha, beta, and gamma) because they do not occur spontaneously and have been associated with the capacity for concentration and preparation to execute an activity, underscoring their importance in the cognitive processes underlying optimal performance or “peak performance” [20].

Because SCPs regulate the excitation threshold, they can be used in self-regulation training when excitation thresholds are altered [24]. To date, no studies have reported SCP training protocols and their impact on performance [16], particularly in a precision sport such as golf. Most studies have prioritized this technique in the clinical field, demonstrating its impact on conditions such as epilepsy, ADHD, and migraines, showing both a reduction in core symptoms and improvement in cognitive variables [25].

Among the most common measures used to evaluate the impact of SCP neurofeedback training are peak frequency, which analyzes the dominant oscillation (Hz) within a frequency band [26]. This is an indicator of neuronal synchronization and improved cognitive and motor processing, as well as greater temporal precision and faster responses [27]. On the other hand, absolute power is used as a measure of total energy within a specific frequency band, measured in μV^2^/Hz, and is associated with neural efficiency when a reduction in cerebral energy expenditure is achieved, along with cognitive or motor efficiency [28].

There is evidence that improved modulation of the beta band (13–30 Hz) is linked to greater neural efficiency and better motor and cognitive performance parameters [29]. Excess beta activity, however, is associated with impulsivity, emotional and attentional dysregulation, and disruptive behavior [30]. When it fails to decrease appropriately, it increases motor errors because of adaptive inefficiency [31]. Specifically, Beta 1 (13–15 Hz) is associated with the sensorimotor rhythm (SMR), particularly when recorded over sensorimotor areas (C3, C4, Cz) [32]. Beta 2 (16–20 Hz) is linked to tasks requiring active attention, visual information processing, and cognitive control [33], and Beta 3 (21–30 Hz, high beta) is related to sustained alertness, visual attention, and, in general, a heightened state of central nervous system activation [34], However, excessive power in this band at rest is associated with increased cortical energy expenditure and inefficient use of neuronal resources [35].

Despite the relevance of these oscillations in achieving neural efficiency and their potential link with enhanced sports performance, their study in sports contexts—particularly in golf—is limited. Therefore, the objective of this study was to evaluate the effect of SCP neurofeedback training on the modulation of beta wave activity in a group of golfers.

## 2. Materials and Methods

### 2.1. Participants and Study Design

A total of 42 amateur golfers over the age of 18 participated in this study. The sample included players with diverse levels of competitive experience. Handicaps ranged widely, from professional status (PRO) to negative values (−3, −1) and high handicaps up to 35, thereby encompassing elite, advanced, and amateur golfers. To ensure comparability and balance, a stratified randomization method was used based on the players’ handicap level. Within each stratum, participants were randomly allocated to either the control or intervention group using a sequence generated in Randomizer.org. The allocation list was prepared by a team member not involved in assessment or participant contact, ensuring that both participants and researchers conducting assessments remained blind to group assignment.

Participant enrolment was conducted by a research assistant who did not have access to the allocation sequence. The allocation was fully concealed until group assignment was completed. Outcome assessors remained blinded to group assignment throughout the study, while participants could not be blinded because the control group was placed on a wait list, constituting a methodological limitation.

This study was conducted as an exploratory randomized controlled trial with a superiority framework, with attention to concerns regarding feasibility, such as the recruitment of a proper sample size. Although the study was conducted in compliance with the CONSORT guidelines, it lacked registration in a public clinical trials register due to its exploratory nature.

Eligibility was based on the following inclusion criteria: being an active golf player, willingness to participate, and provision of signed informed consent. Exclusion criteria were established in accordance with previous reports [36]: (1) changes in the dosage of any psychoactive medication within the 2 months prior to the intervention; (2) required use of benzodiazepines or hypnotic sleep aids; (3) current treatment for bipolar disorder, schizophrenia, schizoaffective disorder, or other psychotic disorders; (4) any significant medical condition interfering with task completion; (5) use of medications affecting hypothalamic–pituitary–adrenal (HPA) axis function or the central nervous system.

**Figure 1:**
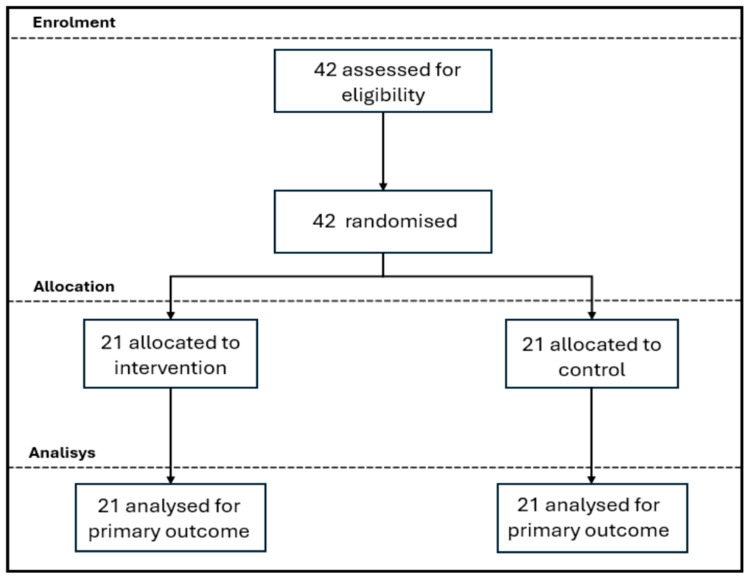
Flowchart illustrating the experimental research procedure.

For comparing the effect of the intervention, both the control and the intervention groups underwent evaluation of slow cortical potentials (SCPs) at baseline (pre-intervention) and post-intervention (Figure 1). The full characterization and evaluation procedure lasted 8 weeks, from July to August 2024. One player was assessed per day (Monday to Friday, 8:00–11:00 a.m., local time, Santiago, Chile) at the designated golf sports facility. Participants assigned to the control group were placed on a waiting list and were offered the intervention once the study was completed. They completed all the measures as the experimental group in the same timeline, but did not receive SCP intervention. All participants performed their usual golf routines and received no additional instructions during the study.

The primary outcome was the determination of changes in beta brain waves in response to slow cortical potential neurofeedback. Secondary outcomes included variations in activation and inhibition ability.

Sample size was calculated with an a priori analysis using G-Power 3.1.9.7. To that end, the primary comparison of interest was the difference between control and intervention groups at the post-intervention assessment, and a two-tailed independent samples test was conducted with α = 0.05, statistical power of 70% and effect size of Cohen’s d = 0.8 [37]. Based on the previously stated, the required sample size was estimated to be 21 participants per group; therefore, 42 participants in total. Although the power is lower than the conventional 80% benchmark, it was selected based on the exploratory nature of this study and was considered sufficient to address the primary research question. No interim analyses or stopping guidelines were planned, given the exploratory and pilot nature of the trial.

### 2.2. Slow Cortical Potential Determination

Baseline SCPs were recorded using ProComp^®^ hardware and Infiniti^®^ software, (Version 6.9.0.0; Thought Technology Ltd., Montreal, QC, Canada). which were used to measure the ability to voluntarily activate (negative) or deactivate (positive) neural activity without visual feedback. A total of 10 trials (5 negative + 5 positive) were conducted, in which the player was required to voluntarily generate the synchronization of excitatory potentials because of the activity of large neuronal assemblies involved in the planning and execution of goal-directed behaviors (focus). Likewise, participants had to voluntarily inhibit and reduce the activity of these excitatory potentials (deactivation), as an outcome of voluntary regulation (calm). The total duration of the evaluation period was 8 weeks (2 months).

### 2.3. Beta Band Recording and Analysis

Beta band activity was recorded using the qEEG Freedom 24-channel system [38]. This device records 19 channels (F8, C3, T5, and O2) according to the international 10–20 electrode placement system on the scalp [39], and was processed with BrainAvatar software (Version 4.7.5.844; BrainMaster Technologies Inc., Bedford, OH, USA) [40]. Measurements were performed both pre- and post-intervention during the 8-week evaluation period.

Recordings of beta band activity were obtained using the qEEG Freedom 24-channel system [38] with electrodes positioned at F8, C3, T5, and O2, following the international 10–20 system [39], and analyzed with BrainAvatar software (BrainMaster Technology^®^) [40]. To assess training-related changes, we focused on these four electrode sites given their functional relevance to the neural processes underpinning golf performance. C3, located over the left sensorimotor cortex, is central to fine motor execution and has consistently demonstrated task-related beta modulation during movement preparation and control [41]. O2, over the right occipital cortex, reflects visual processing and visuomotor integration, both essential for accurate target alignment and perceptual strategies such as the “quiet eye” in precision sports [42]. F8, in the right prefrontal cortex, is associated with inhibitory control and emotional regulation, processes critical for maintaining performance under competitive pressure and reducing the risk of performance breakdowns [43,44]. Finally, T5, located in the left temporoparietal region, supports visuospatial integration and contextual memory, facilitating adaptive spatial representations required for shot planning and execution [45,46]. Collectively, these sites encompass motor, visual, executive, and integrative functions, providing a comprehensive neurophysiological framework for evaluating SCP-induced modulation of beta oscillations within the neural efficiency model in golf. This was performed pre- and post-intervention, and the analysis was carried out using normative references by age and sex [47] for the eyes-open resting-state condition. For the analysis, peak frequency was used with the purpose of analyzing the exact point within the beta band (Hz) where the energy reaches its maximum oscillatory power [27]. That is, this analysis allowed us to determine the frequency with maximum power within a band and to identify the dominant rhythm and its precision for a given activity, in this case, eyes-open resting-state. On the other hand, absolute power was analyzed as a measure of the total energy within the beta band (μV^2^/Hz), which allowed us to obtain information about how much activation (electrical activity) exists in beta [27], serving as a measure to quantify the level of cortical activation associated with a specific activity.

### 2.4. Intervention

The SCP neurofeedback intervention was implemented in accordance with established protocols reported in studies [29]. Participants completed 16 training sessions, each consisting of 4 blocks of 40 trials (160 trials per session), resulting in a total of 2560 trials. The protocol was designed to train players to voluntarily generate both negative and positive shifts in slow cortical potentials. Negative shifts have been shown to reflect cortical activation [48], whereas positive shifts indicate cortical inhibition [22]. Cortical activation is associated with an increased allocation of physiological resources, thereby facilitating processes such as attention and cognitive functioning. In contrast, cortical inhibition is linked to reduced cortical excitability, contributing to the regulation and calming of mental activity. Overall, the capacity to voluntarily modulate SCPs in either direction is considered a key mechanism for enhancing athletes’ self-regulation abilities, which may translate into improved performance in precision-demanding sports.

The presence of any adverse events or harms was assessed non-systematically throughout the study. Participants were instructed to report any unexpected or unwanted symptoms they experienced during the training sessions or post-intervention.

### 2.5. Electrode Montage

SCP neurofeedback recordings were obtained from the vertex (Cz), a standard site for monitoring slow cortical potentials given its central location over the supplementary motor and sensorimotor cortices, which are strongly implicated in motor preparation and self-regulation processes [23,49]. To control for ocular artifacts, three electrodes were placed to record vertical and horizontal electrooculogram (EOG), a procedure widely used to minimize contamination from eye blinks and saccades in SCP and EEG recordings [50]. An additional electrode served as ground. To prevent slow potential drifts, sintered silver/silver chloride (Ag/AgCl) electrodes filled with conductive paste were employed, as they provide stable long-term recordings with low polarization [51]. Impedances were maintained below 5 kΩ, preparing the skin with an abrasive cleaning paste, following established guidelines for clinical and research EEG [52].

### 2.6. Signal Processing

SCP self-regulation training required monitoring of two different brain states. First, the player was asked to reduce the excitation threshold (i.e., to evoke a negative shift) or to increase it (i.e., to produce a positive shift). Due to the continuous change of SCPs from negative to positive, as well as for practical reasons, each trial lasted approximately 8 s, preceded by a passive phase of about 2 s. During the passive phase, a baseline was calculated over a certain number of milliseconds. The mean amplitude of the passive phase was set to zero as the baseline for the subsequent active phase. Feedback was provided through a visual symbol (upward or downward arrow) and a rectangle (top of the screen), simultaneously reflecting and controlling muscle tension (noise).

Every 62.5 milliseconds, SCP amplitude was calculated as an average of the previous 500 milliseconds. The position of the feedback signal (in this case, the arrow) indicated the difference in each 500-millisecond amplitude compared to the baseline. For example, the signal could move upward as a negative shift was produced, while downward movements indicated that the SCP was less negative than during the baseline; that is, cortical positivity (negativity was inhibited) was being produced.

### 2.7. Procedure

As previously mentioned, SCP training included two tasks: producing negative shifts and producing positive shifts. Participants were asked to look at a screen and, based on the arrow displayed, respond by activating or deactivating their own neural activity. Each trial lasted 18 s: 8 s to attempt to activate neural activity (negative potential, upward arrow), 2 s of rest (baseline), and 8 s of voluntary deactivation of neural activity (positive potential, downward arrow).

In addition to feedback trials (visual feedback), transfer trials without visual feedback were also presented. During the third block of the 40 trials, 10 trials (5 activation and 5 deactivation) were presented without feedback (blank screen, no changes), with the purpose of enhancing transfer. Only at the end of each transfer trial was the player informed whether they had succeeded. This feedback was provided by displaying a smiling face representing positive reinforcement when the player had achieved the trial objective.

### 2.8. Sessions

**Figure 2:**
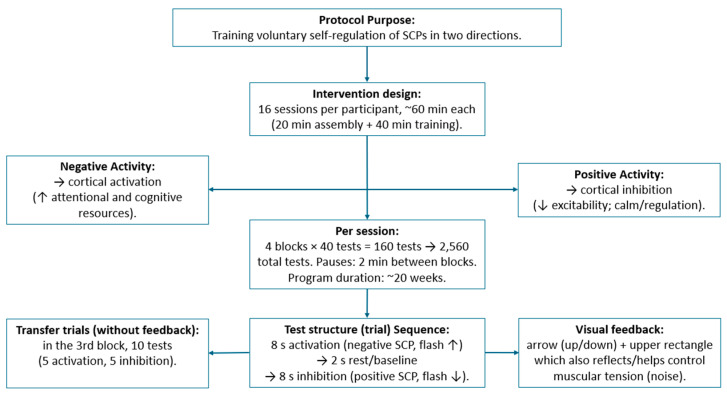
Schematic overview of the SCP self-regulation protocol, including intervention design, trial sequence, and feedback components.

An introductory session was conducted to familiarize participants with the environment and training (Figure 2), and all training sessions were conducted by an expert psychologist certified in neuro- and bio-feedback, responsible for intervention. The concept of efficiency in activating and deactivating neural activity (calm and focus) was introduced as a central component of motor execution. Subsequently, the details of the equipment, sensors, and software were presented. To prevent artifacts, these were demonstrated before training began, allowing players to observe screen movements while being encouraged to intentionally create muscle noise artifacts (e.g., moving the tongue, clenching the jaw, tensing shoulder muscles, or frowning). After these small “experiments,” it was explained that artifacts may be mistaken for successful performance but hinder the learning of self-regulation skills.

Training sessions (1 to 16) were conducted in a single golf facility in Santiago, Chile, ensuring consistency among sessions. All sessions lasted 60 min each, consisting of electrode placement and removal (20 min) and SCP neurofeedback training (40 min). In total, 16 sessions were conducted, with 160 trials per session in 4 blocks of 40 trials, including 2 min breaks between blocks, for a total of 2560 trials per participant. The training program lasted 20 weeks for the 21 participants in the intervention group, with 100% adherence.

In accordance with the Cochrane Risk of Bias 2.0 framework (Ros et al., 2020) [18], we considered potential biases related to deviations from the intended intervention and the selection of reported results. Although no formal blinding was implemented and protocol deviations were not systematically monitored due to the small sample size and exploratory nature of the study, all sessions were supervised by the same research team to minimize deviations, and all pre-specified outcomes were reported to reduce the risk of selective reporting.

### 2.9. Statistical Analysis

A baseline characterization of participants was performed, and demographic information was summarized. SCP results and beta activity were recorded before and after the intervention. A similar distribution of handicap was observed across groups; however, this factor was not controlled, given the low number of participants, which limited the feasibility of including handicap as a covariate in the analyses. The results were first assessed for normality. When the assumption of normality was met, group comparisons were conducted using Student’s t-tests. In cases where this assumption was not satisfied, non-parametric alternatives, including Mann–Whitney U and Wilcoxon tests, were applied to evaluate the effects of SCP neurofeedback training on beta wave activity. Effect size was determined using rank biserial correlation (RBC). All participants completed the study, and no missing data were observed. Therefore, no imputation methods were required. For all statistical analyses, the significance level was set at *p* < 0.05. Data are presented as median and interquartile range (IQR). Data analysis was carried out using Python software (Python Software Foundation. (2024). Python (Version 3.13.5)).

## 3. Results

### 3.1. Demographic Characteristics

Both the training and control groups comprised 21 participants each. Gender distribution was similar across groups, with males representing the majority in both (76.2% in the control group and 71.4% in the training group).

### 3.2. Slow Cortical Potentials

**Figure 3:**
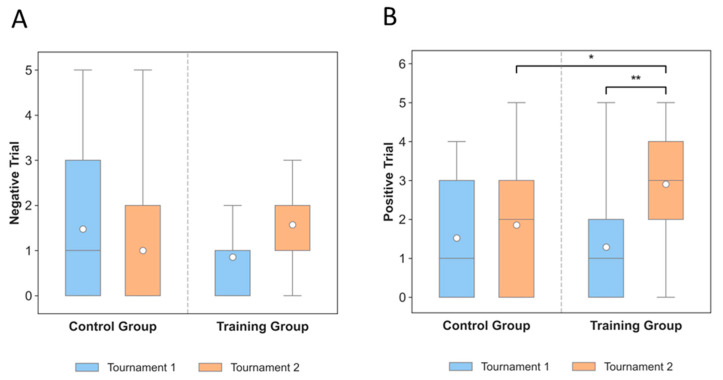
Distribution of SCP activity across experimental conditions. In tournaments 1 and 2 (pre-post), each participant performed 120 putts, divided into 4 sets of 30 shots from different distances (4 m, 3 m, 2 m, and 1 m) on one hole of the golf course. (**A**) Negative trials; (**B**) Positive trials (*p* = 0.0024 and 0.0328). *n* = 21. In (**B**), the asterisks indicate statistically significant differences between conditions. A single asterisk (*) denotes a significance level of *p* < 0.05, while a double asterisk (**) indicates a more robust significance level of *p* < 0.01. These results suggest that the differences in positive SCP trials between tournaments and groups were statistically meaningful, with the training group exhibiting stronger modulation effects after SCP neurofeedback.

**Figure 4:**
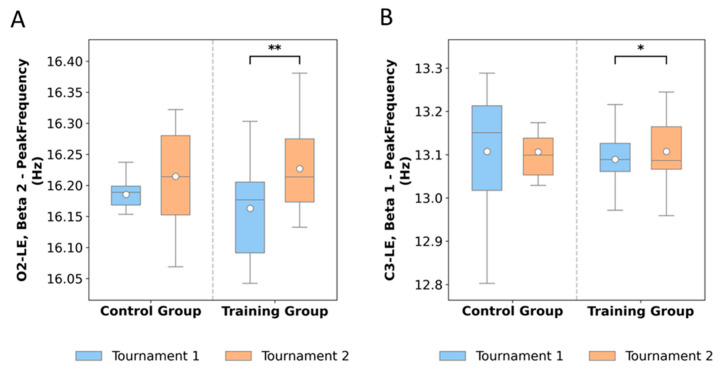
Effects of neurofeedback training on beta oscillations across cortical sites. (**A**) O2-LE Beta 2 peak frequency (*p* = 0.008); (**B**) C3-LE Beta 1 peak frequency (*p* = 0.047). *n* = 21. In Figure, the asterisks denote statistically significant differences between tournaments within the training group. A single asterisk (*) represents a significance level of *p* < 0.05, while a double asterisk (**) indicates a stronger level of significance (*p* < 0.01). These results demonstrate that neurofeedback training induced significant modulation of beta oscillatory activity, particularly at occipital (O2) and central (C3) sites, suggesting enhanced cortical efficiency and stability in the trained participants.

Slow cortical potentials (SCPs) were analyzed under negative and positive trial conditions at baseline and post-intervention (Table 1, Figure 3 and Figure 4). At baseline, median SCP values during negative trials were close to zero in the training group (median = 0, IQR = 1) and slightly higher in controls (median = 1, IQR = 3). Post-intervention, the training group showed an increase in negative trial SCPs (median = 1, IQR = 1), while the control group remained stable (median = 0, IQR = 2).

In positive trials, baseline values were similar between groups (median = 1–2). After training, the intervention group showed a marked increase in SCP amplitudes (median = 3, IQR = 2), whereas controls remained lower (median = 2, IQR = 3).

Statistical analysis showed elevated variability at baseline and post-intervention, with higher post-training amplitudes visible in the positive trial condition (Figure 3B). Positive trials significantly increased from pre- to post-training in the intervention group (*p* = 0.0024, RBC = 0.79), reflecting a large effect size and indicating a consistent post-intervention enhancement. Moreover, direct post-intervention comparisons showed that SCPs were significantly higher in the training group than in the control group (*p* = 0.0328, RBC = 0.38). Collectively, these findings demonstrate that SCP modulation was successfully achieved through training, particularly in the positive trial condition.

### 3.3. Beta Waves

EEG beta oscillations were assessed in terms of peak frequency and absolute power across multiple regions (Table 2, Figure 4). At electrode C3-LE (Beta 1), the training group showed a slight increase in peak frequency from 13.08 Hz pre-training to 13.12 Hz post-training, while controls remained stable (13.10 Hz to 13.09 Hz). At O2-LE (Beta 2), training led to a small increase (16.16 to 16.21 Hz), whereas controls were unchanged. These shifts were minor, with medians remaining close to baseline values, suggesting minimal impact of training on frequency stability.

In contrast, marked differences emerged in frontal and temporal regions. At F8-LE, Beta 2 absolute power decreased from 1.90 to 1.22 following training, while controls increased slightly from 1.82 to 2.03. A similar trend was observed for Beta 3, with training participants decreasing from 4.04 to 2.77, compared to a modest reduction in controls (3.88 to 3.77). Temporal sites also reflected training-related reductions: T5-LE Beta 2 decreased from 2.38 to 1.65, while controls increased from 1.87 to 2.40. For High Beta at T5-LE, training produced a drop from 1.97 to 1.25, whereas controls increased from 1.37 to 1.96. Overall, these patterns indicate a consistent reduction in beta power following training, while control participants maintained or increased beta activity over time.

At the occipital electrode O2-LE, Beta 2 peak frequency (Figure 4A) significantly increased after training (*p* = 0.008, RBC = 0.31), which seems to indicate an enhancement of oscillatory synchronization in the visual cortex.

At the central electrode C3-LE, Beta 1 peak frequency (Figure 4B) exhibited a modest but significant increase after training (*p* = 0.047, RBC = 0.24). This localized enhancement in the motor cortex seems to indicate that the intervention not only reduced beta power in frontal and temporal areas but also facilitated improved oscillatory dynamics in motor-related regions.

**Figure 5:**
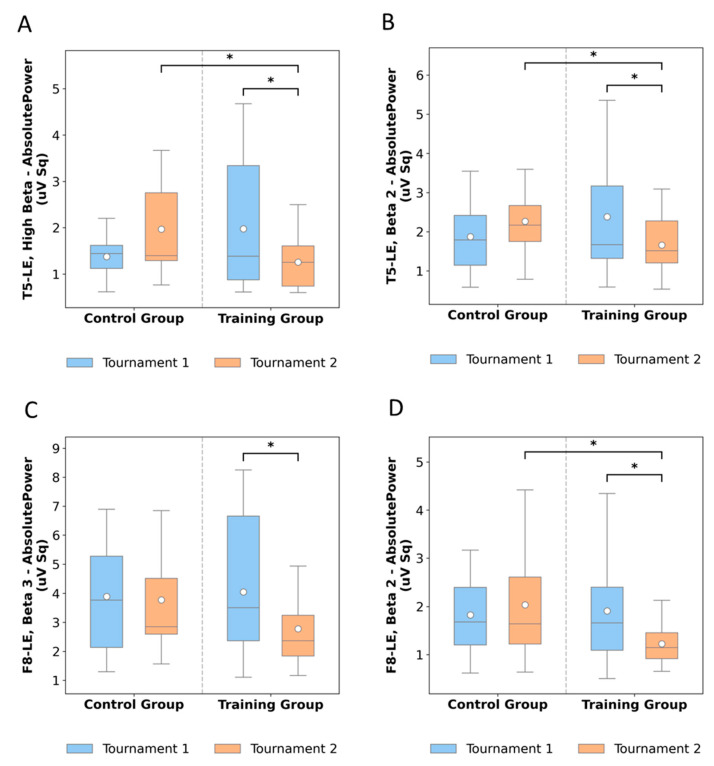
Effects of neurofeedback training on beta oscillations across cortical sites. (**A**) T5-LE High Beta absolute post-training (*p* = 0.040); (**B**) T5-LE Beta 2 absolute power post-training (*p* = 0.016, *p* = 0.027); (**C**) F8-LE Beta 3 absolute power post-training (*p* = 0.040); (**D**) F8-LE Beta 2 absolute power (*p* = 0.044, *p* = 0.035). *n* = 21. In this Figure, asterisks denote statistically significant differences between tournaments and groups. A single asterisk (*) indicates a significance level of *p* < 0.05. These results reveal that neurofeedback training produced meaningful modulations in beta-band absolute power, particularly over temporal (T5) and frontal (F8) cortical regions, suggesting enhanced cortical regulation and adaptive neuroplastic changes associated with training-induced self-regulation.

At the temporal site T5-LE, significant reductions were observed in both High Beta (Figure 5A) and Beta 2 (Figure 5B) absolute power. For High Beta, training produced a marked post-intervention decrease compared to baseline (*p* = 0.039, RBC = −0.57), consistent with suppression of high-frequency beta activity that was not present in controls. Similarly, Beta 2 absolute power (Figure 5B) decreased significantly after training (*p* = 0.016, RBC = −0.64), and direct comparisons between groups confirmed lower values in the training group relative to controls at post-test (*p* = 0.027, RBC = −0.42). These findings suggest that the intervention downregulated temporal beta power, particularly in higher frequency ranges.

In the frontal region, both F8-LE Beta 3 (Figure 5C) and F8-LE Beta 2 (Figure 5D) absolute power decreased following training. Beta 3 showed a significant pre- to post-training reduction (*p* = 0.040, RBC = −0.57), while Beta 2 also declined (*p* = 0.044, RBC = −0.57), with group comparisons at post-test confirming lower power in the training group compared to controls (*p* = 0.035, RBC = −0.42). These results reinforce that the intervention suppressed excessive beta power in frontal regions.

## 4. Discussion

The present study evaluated the effects of a slow cortical potential (SCP) neurofeedback training program on beta band activity in golfers, analyzing brain function at eyes-open resting state before and after the intervention. The intervention group completed 16 sessions (2560 trials in total), while the control group did not receive training. The results revealed a dual effect of training: a consistent reduction in beta absolute power in frontal and temporal cortices, coupled with increases in peak frequency in motor and occipital regions. This pattern seems to indicate that neurofeedback selectively modulated cortical oscillations, downregulating excessive activation in associative areas while enhancing temporal synchronization within sensorimotor and visual networks.

These findings are consistent with the neural efficiency theory, which posits that expert athletes make more precise and economical use of cerebral resources, reflected in reduced absolute power and greater precision in peak frequency of brain oscillations [53]. The reduction in absolute power observed in temporal and frontal regions (particularly at F8 and T5) could indicate a decreased cortical overload and “neural noise,” favoring more efficient executive control and more precise visuospatial integration. These results align with previous evidence on neurofeedback in sports contexts [54,55]. SMRs (12–15 Hz, C3/Cz/C4) have been successfully used to improve motor response precision [25,29]. The theta/beta protocol (↓4–7 Hz; ↑15–20 Hz) is used to optimize sustained attention and inhibitory control [15,16] and improvements in reaction time, precision skills, and motor performance in athletes and healthy adults [16,17].

A novel finding of this study was the significant increase in the number of positive SCP trials (deactivation) in the training group, which could reflect an enhanced ability for voluntary cortical inhibition. This is a novel finding for golfers and could be attributed to the optimization of the ability to voluntarily inhibit brain activity, as a result of cortical activity regulation [49]. Interestingly, similar results have been observed in archery and rifle shooting, where neurofeedback-based protocols improved the athlete’s ability to suppress unnecessary cortical activation, thereby increasing stability and accuracy under pressure [56]. Likewise, in pianists and violinists, SCP training has been associated with enhanced inhibitory control of cortical activity, allowing performers to maintain precision in highly demanding sequences [57]. These parallel results could suggest that the mechanism observed in golfers reflects a broader capacity for neurofeedback to optimize the balance between cortical excitation and inhibition across activities that demand fine motor control, attentional regulation, and emotional stability.

Regarding peak frequency, the increases observed in motor regions (C3, Beta 1) and visual regions (O2, Beta 2) can be interpreted as micro-adjustments that enhance oscillatory precision without altering the intrinsic resonance of these networks. Such subtle shifts, remaining within normative ranges, have been described in studies of motor and visual performance as indicators of improved functional synchronization [47,58]. Nevertheless, these micro-changes must be interpreted cautiously, especially in eyes-open resting conditions where contextual factors may influence oscillatory variability [59].

Conversely, the reduction in absolute power observed at F8 (Beta 2 and Beta 3) and T5 (Beta 2 and High Beta) suggests an improved executive regulation and visuospatial integration through the attenuation of cortical overload. This partially contrasts with studies reporting increases in absolute power following neurofeedback, highlighting that outcomes are strongly influenced by the cognitive strategies employed by participants during training rather than reflecting a single universal mechanism of cortical reorganization [19,60].

Overall, our findings extend prior evidence by demonstrating that SCP neurofeedback not only enhances self-regulation in clinical populations [49] but also in high-performance contexts such as golf. The enhanced capacity for cortical inhibition observed here parallels results from other precision-demanding populations, including marksmen [56], surgeons [61], and pilots [62], all of whom showed improved cognitive control and reduced cortical activation after neurofeedback. This convergence suggests that SCP-based training may represent a cross-domain mechanism to optimize neural efficiency in individuals exposed to conditions of high cognitive–motor demand.

Taken together, the results support the hypothesis that SCP neurofeedback can enhance neural efficiency in golf by reducing cortical overload and improving the temporal precision of beta oscillations. Future studies should compare different athletic populations (e.g., golfers, shooters, archers, tennis players) to determine whether these effects are sport-specific or generalizable. Additionally, longitudinal research is needed to assess the sustainability of these changes and their impact on competitive performance.

## 5. Limitations

The limitations of our study include several methodological and conceptual factors. First, the relatively small sample size limited the statistical power of our analyses and restricted the possibility of including handicap as a covariate or stratification variable, which could have provided a more nuanced interpretation of the results. Second, the wide confidence intervals observed in some measures indicate variability that reduces the precision of our estimates. Third, the lack of blinding may have introduced bias in both data collection and interpretation. In addition, the generalizability of resting-state EEG findings to actual task performance remains limited, as neural activity during rest may not fully capture the dynamics involved in real-time cognitive or affective processes. Finally, the absence of hormonal measures during performance precludes examination of the potential modulatory role of endocrine factors, which could be particularly relevant given the interplay between neurophysiological and hormonal systems.

## 6. Conclusions

The present study provides novel evidence that slow cortical potential (SCP) neurofeedback could effectively modulate beta band activity in competitive golfers. In fact, training led to a dual effect: a reduction in absolute beta power in frontal and temporal regions and a slight increase in peak frequency within motor and occipital areas. This spectral pattern is consistent with the neural efficiency theory, suggesting that athletes who optimize cortical resource allocation achieve lower energetic costs and greater temporal precision in neural processing.

Importantly, the significant increase in positive SCP trials could indicate that the golfers’ sample developed an improved capacity for voluntary cortical inhibition, a mechanism essential for regulating attentional, motor, and emotional demands under competitive conditions. This finding aligns with evidence from other precision-demanding domains such as archery, shooting, and music, reinforcing the role of SCP neurofeedback as a tool for enhancing inhibitory control and self-regulation.

In conclusion, these results highlight the potential of SCP training to support sport performance optimization, particularly in disciplines where fine motor execution, visuospatial integration, and emotional stability are critical. Beyond golf, the application of SCP neurofeedback could extend to athletes across sports and cultures, contributing to performance enhancement through the promotion of neural efficiency. Future research should expand to larger and more diverse cohorts and assess the sustainability of training effects over time, paving the way for the integration of SCP protocols into evidence-based sport neuroscience interventions.

## Figures and Tables

**Table 1:** Baseline SCP.

Group	Variable	Mean	SD	Median	IQR
Pre-Training	Negative Trial	0.85	1.55	0	1
Pre-Control	Negative Trial	1.47	1.69	1	3
Post-Training	Negative Trial	1.57	1.46	1	1
Post-Control	Negative Trial	1.00	1.54	0	2
Pre-Training	Positive Trial	1.28	1.61	1	2
Pre-Control	Positive Trial	1.52	1.47	1	3
Post-Training	Positive Trial	2.90	1.33	3	2
Post-Control	Positive Trial	1.85	1.68	2	3

**Table 2:** Beta wave activity across cortical regions.

Group	Variable	Mean	SD	Median	IQR
Pre-Training	C3-LE, Beta 1-Peak Frequency	13.08	0.06	13.08	0.06
Post-Training	C3-LE, Beta 1-Peak Frequency	13.12	0.11	13.08	0.11
Pre-Control	C3-LE, Beta 1-Peak Frequency	13.10	0.14	13.15	0.19
Post-Control	C3-LE, Beta 1-Peak Frequency	13.09	0.18	13.09	0.11
Pre-Training	O2-LE, Beta 2-Peak Frequency	16.16	0.07	16.17	0.11
Post-Training	O2-LE, Beta 2-Peak Frequency	16.21	0.08	16.20	0.10
Pre-Control	O2-LE, Beta 2-Peak Frequency	16.20	0.07	16.19	0.04
Post-Control	O2-LE, Beta 2-Peak Frequency	16.21	0.07	16.21	0.12
Pre-Training	F8-LE, Beta 2-Absolute Power	1.90	1.19	1.65	1.31
Post-Training	F8-LE, Beta 2-Absolute Power	1.22	0.42	1.14	0.53
Pre-Control	F8-LE, Beta 2-Absolute Power	1.82	0.79	1.67	1.19
Post-Control	F8-LE, Beta 2-Absolute Power	2.03	1.22	1.63	1.38
Pre-Training	F8-LE, Beta 3-Absolute Power	4.04	2.21	3.50	4.29
Post-Training	F8-LE, Beta 3-Absolute Power	2.77	1.64	2.36	1.40
Pre-Control	F8-LE, Beta 3-Absolute Power	3.88	1.83	3.76	3.14
Post-Control	F8-LE, Beta 3-Absolute Power	3.77	2.01	2.85	1.91
Pre-Training	T5-LE, Beta 2-Absolute Power	2.38	1.52	1.66	1.84
Post-Training	T5-LE, Beta 2-Absolute Power	1.65	0.78	1.51	1.07
Pre-Control	T5-LE, Beta 2-Absolute Power	1.87	0.86	1.79	1.26
Post-Control	T5-LE, Beta 2-Absolute Power	2.40	1.01	2.20	0.97
Pre-Training	T5-LE, High Beta-Absolute Power	1.97	1.34	1.38	2.46
Post-Training	T5-LE, High Beta-Absolute Power	1.25	0.57	1.25	0.86
Pre-Control	T5-LE, High Beta-Absolute Power	1.37	0.40	1.44	0.49
Post-Control	T5-LE, High Beta-Absolute Power	1.96	1.02	1.39	1.46

## Data Availability

Data will be available by request to the corresponding author.

## Abbreviation

The following abbreviation is used in this manuscript:

SCP	Slow cortical potential
